# Cometabolic regulation of glucose and glycerol to enhance 1,3-propanediol yield by *Clostridium butyricum*

**DOI:** 10.1186/s12934-026-02971-6

**Published:** 2026-03-08

**Authors:** Li Wang, Yaqin Sun, Zhilong Xiu

**Affiliations:** MOE Key Laboratory of Intelligent Bio-Manufacturing, School of Bioengineering, Dalian University of Technology, Dalian, 116024 China

**Keywords:** 1,3-Propanediol, *Clostridium butyricum*, Cometabolism, Regulatory mechanism

## Abstract

**Background:**

1,3-Propanediol (PDO) is a high value-added product with significant potential for application and development in various industries including textiles, cosmetics, food, and chemicals. Microbial fermentation for 1,3-PDO production has advantage over chemical synthesis in response to environmental and energy challenges. In view of the complexity and benefits of biometabolic synthesis, there is a necessity to enhance the yield of 1,3-PDO to glycerol by means of co-substrates, whilst concomitantly reducing the cost of production.

**Results:**

In this study, the efficiency and cost-effectiveness of 1,3-PDO synthesis by *Clostridium butyricum* DL07 were improved through elucidating the regulatory mechanism of glucose-glycerol cometabolism and optimizing the ratio of co-substrates. Among the screened co-substrates, glucose emerged as the optimal choice for enhancing the yield and concentration of 1,3-PDO synthesized from glycerol. If the molar ratio of glucose to glycerol was 0.51 mol/mol, the 1,3-PDO yield reached 0.90 mol/mol in the co-substrate fermentation. Real-time quantitative PCR revealed that the *dha* operon expression was affected by glucose on inhibition of GDH (*dhaD*) and phasically upregulation of PDOR activity (*dhaT*). Stoichiometric analysis combined with further optimization of the different feeding strategies increased the 1,3-PDO yield to the highest level of fed-batch fermentation (0.86 mol/mol) reported to date for natural strains. The material cost analysis demonstrated that the co-substrate strategy reduced raw material cost by 13–20%.

**Conclusions:**

This study presents an investigation into the synthesis of 1,3-PDO through cometabolism in *C. butyricum* DL07. The research elucidates the synergistic impact of co-substrate ratios and temporal regulation on the synthesis of 1,3-PDO. Moreover, it offers a theoretical basis and a practical approach for achieving cost-effective and efficient industrial production.

**Supplementary Information:**

The online version contains supplementary material available at 10.1186/s12934-026-02971-6.

## Background

In recent years, the global emphasis on sustainable development has accelerated the transition from petrochemical-based chemicals to bio-based alternatives, with the market value projected to exceed $270 billion by 2032 [1]. Biodiesel, an environmentally friendly, renewable, and biodegradable substitute for petrochemical diesel, is derived from renewable biomass resources such as vegetable oils and animal fats, and its production has increased steadily [2]. As a by-product of biodiesel synthesis, crude glycerol is obtained with a yield of 5–10 gallons per 100 gallons of biodiesel produced [3]. The accumulation of excessive glycerol poses environmental risks, disposal challenges, and economic burdens. Consequently, the valorization of glycerol into high-value-added compounds has emerged as a critical research focus [4, 5].

Microbial transformation represents an eco-friendly approach for the biosynthesis of value-added compounds from renewable substrates [6, 7]. This technology has garnered significant research attention due to its high substrate specificity, sustainable nature, and mild reaction conditions, which contrast sharply with conventional chemical synthesis methods that often entail high energy consumption, expensive catalysts, and poor reaction selectivity. Glycerol, in particular, serves as an excellent microbial substrate for producing high-value compounds, including dihydroxyacetone, succinic acid, butanol, 3-hydroxypropionic acid, and 1,3-PDO [8]. Among these, 1,3-PDO is a versatile platform chemical widely utilized in textiles, cosmetics, food additives, and specialty polymers. In particular, the polyester fiber polytrimethylene terephthalate (PTT), synthesized using 1,3-PDO as one of monomers, exhibits excellent properties such as high stretchability, resistance to pollution, and ease of dyeing [9, 10]. Given its broad utility, 1,3-PDO holds substantial market potential, with projections indicating a compound annual growth rate (CAGR) of 10.98% and an estimated market value of $1.63 billion by 2030 [11].

Natural strains known to metabolize glycerol to 1,3-PDO include *Clostridium*, *Klebsiella*, *Citrobacter*, and *Lactobacillus*, among others (Table 1). *K. pneumoniae* and *C. butyricum* are the dominant strains capable of synthesizing 1,3-PDO at high concentrations and yields [12]. However, *K. pneumoniae* is primarily a facultative pathogen and limited in its industrial application. Additionally, it synthesizes numerous by-products, such as 2,3-butanediol, ethanol, and lactic acid, which leads to inefficient carbon utilization and increases the complexity of downstream isolation and purification [13]. In contrast, *C. butyricum* produces fewer fermentation byproducts, and its primary byproduct, butyric acid, possesses a high price and more promising industrial applications. Additionally, it exhibits stable growth and resistance to environmental stresses [14–16]. The *C. butyricum* DL07, identified during our laboratory screening, is a naturally high-yield anaerobic strain capable of producing 1,3-PDO. This strain exhibits minimal metabolic by-products, notably the absence of solvent by-products, offering the benefit of the subsequent separation process [17].

**Table 1:** Production of 1,3-PDO from glycerol by native microorganisms

Microorganism	Fermentation mode	C_1,3−PDO_ (g/L)	Y_1,3−PDO_ (mol/mol)	Q_1,3−PDO_ (g/L/h)	Reference
*C. butyricum* DSM 5431	Batch	56.00	-	1.93	[18]
*C. butyricum* DL07	Fed-batch	104.80	0.65	3.38	[17]
*C. butyricum* AKR102a	Fed-batch	93.70	0.63	3.30	[19]
*C. butyricum* L4	Fed-batch	70.10	0.65	0.47	[5]
*C. butyricum* VPI 1718	Fed-batch	70.80	0.67	0.71	[20]
*K. pneumoniae* DSM 4799	Fed-batch	80.20	0.55	1.16	[21]
*K. pneumonia* ATCC 8724	Fed-batch	62.72	0.73	1.74	[22]
*K. pneumoniae* LX3	Repeated fed-batch	66.00	0.61	3.30	[23]
*C. pasteurianum* G8	Fed-batch	90.55	0.62	3.62	[24]
*C. freundii* VK-19	Fed-batch	47.40	0.53	1.01	[25]
*C. freundii* FMCC-B 294	Fed-batch	68.10	0.48	0.79	[26]
*C. perfringens* GYL	Fed-batch	40.00	0.68	2.00	[27]
*C. diolis* DSM15410	Fed-batch	63.50	-	1.35	[28]
*K. oxytoca* FMCC-197	Fed-batch	50.10	-	0.55	[29]

**Fig. 1:**
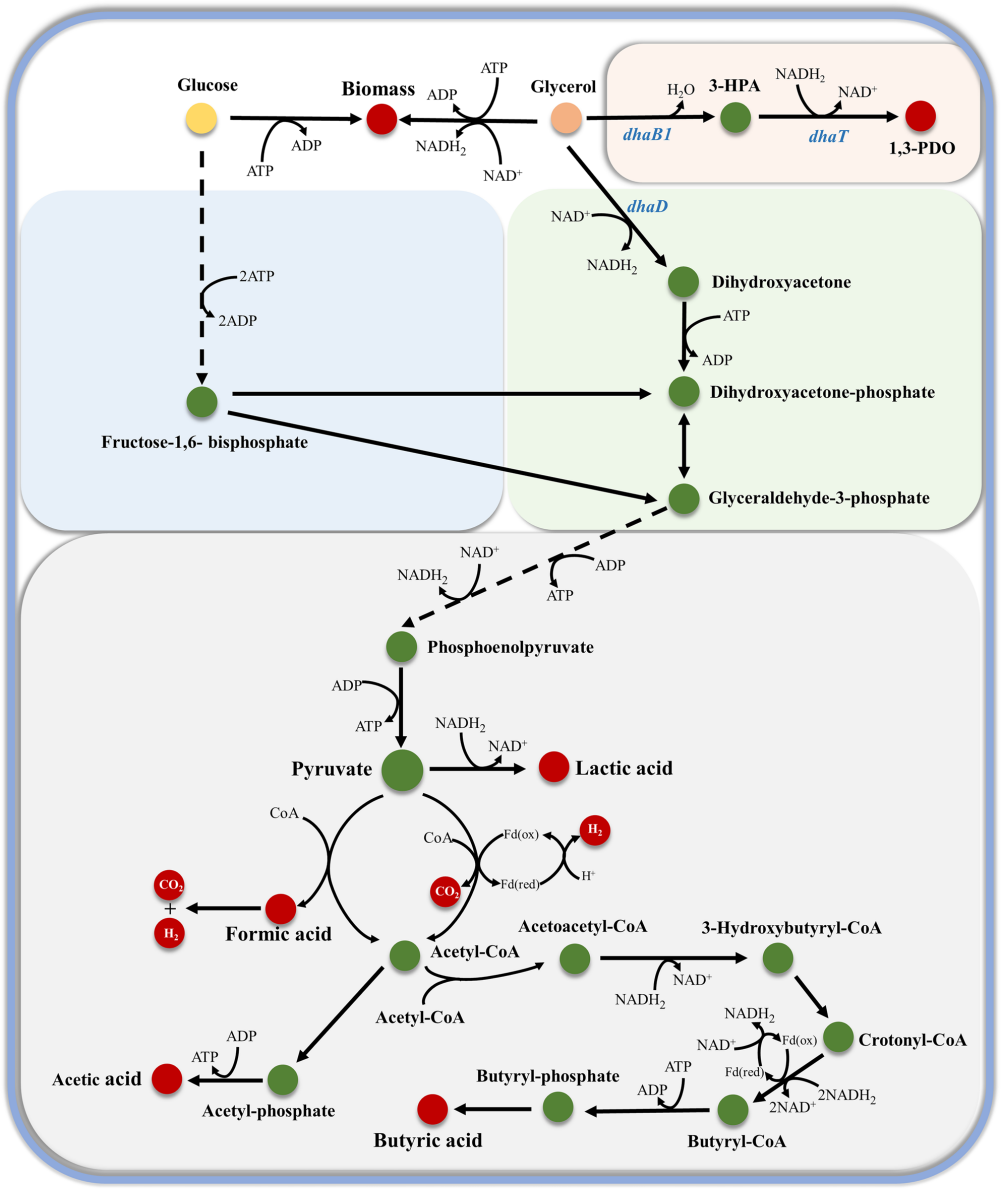
Pathways of glucose and glycerol cometabolism in *C. butyricum*. Notation: substrates (yellow dot and orange dot), intracellular metabolites (green dots), extracellular products (red dots). Individual segments of the glycerol oxidation pathway (green background), the glycerol reductive pathway (pink background), individual segments of the glucose metabolic pathway (blue background), and the metabolic pathways shared by glucose and glycerol (gray background). Key genes regulated by *dha* regulon: *dhaD*: glycerol dehydrogenase; *dhaB1*: glycerol dehydratase; *dhaT*: 1,3-propanediol oxidoreductase

Current microbial bioconversion pathways of glycerol face a fundamental limitation due to the branched metabolic architecture of glycerol distribution. This structural configuration induces carbon flux competition between oxidative and reductive branches, particularly during 1,3-PDO biosynthesis as shown in Fig. 1. The redox imbalance limits the theoretical maximum 1,3-PDO yield to be 0.72 mol/mol due to insufficient NADH regeneration [30]. For example, *C. butyricum* VPI 1718 was used to produce 67.9 g/L of 1,3-PDO at a yield of 0.67 mol/mol in a fed-batch fermentation under non-sterile conditions, and 70.8 g/L of 1,3-PDO with a yield of 0.67 mol/mol at 100 h under continuous nitrogen supply [20, 31].

It is usually regarded that the presence of glucose in the fermentation medium severely suppresses glycerol uptake and metabolism via the carbon catabolite repression (CCR). However, co-substrate fermentation employing hexose/pentose has been proven to be effective in supplying essential ATP and NADH for cellular metabolism via the pentose phosphate pathway (PPP) as shown in Fig. 1. For examples, *K. pneumoniae* ME-303 used hemicellulosic hydrolysates as co-substrate to fermente glycerol producing 71.58 g/L of 1,3-PDO at a yield of 0.65 mol/mol and a productivity of 1.93 g/L/h, which were 17.8%, 25.0%, and 17.7% higher than those of glycerol alone [32]. Similarly, *C. butyricum* demonstrated a 50% enhancement in 1,3-PDO productivity when utilizing cassava pulp as a co-substrate with glycerol [33]. Additionally, the 1,3-PDO yield of 0.88 mol/mol was achieved using *C. butyricum* L4 at a glycerol-to-glucose ratio of 10:1. If glucose was excessively added, this metabolic benefit was substantially compromised by CCR, which is a regulatory mechanism favoring preferential utilization of readily metabolizable substrates like glucose, thereby diminishing glycerol uptake and conversion capacity [5]. Although the use of sugar-based substrates promotes NADH production, their potential inhibitory effect also poses a challenge to glycerol metabolism. In fact, determining the limits of sugar-based substrate addition is critical for co-fermentation with glycerol to maximize 1,3-PDO output. Taking *K. pneumoniae* as an example, if glycerol was used only to produce 1,3-PDO, the required maximum ratio of glucose to glycerol was 0.32 mol/mol under anaerobic conditions, and can be reduced under microaerobic conditions [34]. In order to design a scientific and rational co-substrate fermentation, it is necessary to carry out a theoretical stoichiometric analysis of the cometabolism of glycerol and sugars, aiming to avoid the accumulation of by-products from excessive carbon sources, to achieve the maximum redirection of the metabolic flow, and to provide an important guiding basis for the process scale-up.

This study aimed to develop an economically viable 1,3-PDO production process through co-substrate fermentation using *C. butyricum* DL07. Initially, different sugars were evaluated, especially the metabolic pathways of xylose and L-arabinose were examined. Subsequently, the mechanism of glucose and glycerol cometabolism was investigated through batch fermentations. Furthermore, stoichiometric analysis was employed to guide co-substrate fed-batch fermentations, assessing the potential for co-substrate strategies in industrial settings.

## Methods

### Material

All chemicals and equipment were obtained from commercial suppliers as follows: yeast extract FM888 (Angel Yeast Co., Ltd., Hubei, China), calcium carbonate and citric acid (Sinopharm Chemical Reagent Co., Ltd., Shanghai, China). Glycerol and other medium components were acquired from Damao Chemical Reagent Partnership Enterprise (Tianjin, China). Fermentation equipments include a YZ151X peristaltic pump (LongerPump Co., Ltd., Hebei, China) and a bioreactor with automated pH control and feeding system (Baoxing Bio-Engineering Equipment Co., Ltd., Shanghai, China).

### Strain, media, and culture conditions

*C. butyricum* DL07 was isolated from activated sludge in an anaerobic digester and deposited in our lab. The composition of the seed medium followed Zhou et al. [35], while the fermentation medium was prepared according to Jiang et al. [36]. The strain was preserved in seed culture medium with 20% (v/v) glycerol at -80 °C. The strain was activated in the seed medium at 4% (v/v), 37 °C and 200 rpm for 12 h. After activation, 2% (v/v) of the broth was transferred into fresh seed medium for further inoculation at the OD_650_ value in the range of 2.8–3.2. The strain activation and seed cultivation were carried out in a total of 100 mL of seed culture medium deoxygenated in 250 mL anaerobic serum flasks by sparging N_2_ (99.9%) at 400 mL/min for 3 min and then sterilized at 121 °C for 20 min.

### Co-fermentation of sugar and glycerol in bioreactors

Batch fermentations were conducted in a 5.0 L bioreactor containing 1.8 L of sterile fermentation medium at a glycerol concentration of 40 g/L. The medium was supplemented with 0.2 L of separately sterilized sugar solutions at different concentrations (40, 20, 10, and 5 g/L). N₂ (99.9%) was sparged into the bioreactor at a flow rate of 0.10 vvm for 1 h before and after the start of fermentation to establish an anaerobic environment. A 10% (v/v) inoculum of the mature seed culture was introduced into the 5.0 L bioreactor. The fermentation conditions were maintained at 37 °C and 250 rpm, with the pH automatically controlled at 7.0 using a 5 M NaOH solution. Unlike the batch fermentation, the fed-batch fermentation process was carried out according to Wang et al. [17]. The residual glycerol concentration during fermentation was maintained at approximately 20 g/L through a control-feeding, thereby preventing the growth inhibition due to high glycerol concentrations.

### Real-time quantitative PCR

The appropriate amounts of samples were collected at different fermentation intervals. Total RNA was extracted using the Bacteria RNA Extraction Kit (Vazyme Biotech Co., Ltd. R403). The isolated total RNA was used as a template for reverse transcription to obtain cDNA using HiScript IV All-in-One Ultra RT SuperMix (Vazyme Biotech Co., Ltd., R433) for real-time quantitative PCR. Primers for *dhaB1*, *dhaT*, *dhaD*, and the 16 S rRNA internal reference gene were designed using the online tool (https://store.sangon.com/newPrimerDesign) based on the cDNA sequences. The primer sequences of the above four genes were listed in Table 2. The results were analyzed for relative quantification using SupRealQ Ultra Hunter SYBR qPCR Master Mix (Vazyme Biotech Co., Ltd., Q713) and a CFX Opus 96 Real-Time PCR System (BIO-RAD), in accordance with the manufacturer’s instructions.

**Table 2:** The primer sequences of the four genes

Gene	Forward (5’--3’)	Reverse (5’--3’)
*dhaB1*	CCCAAGAGGCGGAAAATTTCAACC	CTTGACGGAGAAACCCCATCAGC
*dhaT*	AGCAGGATTAACAGCAGCTACAGG	ATTTGCAACTCCATGCGGCATATC
*dhaD*	TGGTCTGGGGTGGTGGCAAGG	CTGAATGGCTTGTTGGTGCATCTG
Internal reference	GGAAGAATACCAGTGGCGAAGGC	CATCGTTTACGGCGTGGACTACC

### Analytical methods

Biomass in the fermentation broth was measured as optical density (OD) at 650 nm using a UV-visible spectroscopy system (UV-5100, Metash Instruments Co. Ltd., Shanghai, China). The substance contents in the fermentation broth, i.e. glycerol, 1,3-PDO, acetic acid, lactic acid, butyric acid, and sugar, were analyzed using high-performance liquid chromatography (HPLC) (Waters, Milford, USA) equipped with an Aminex HPX 87 H column (300 × 7.8 mm) (Bio-Rad, Hercules, CA), a Waters 2414 refractive index detector, and a Waters 2707 autosampler. The fermentation supernatant was obtained by centrifugation of 1 mL of fermentation broth at 12,000 rpm for 10 min to remove the cells. The supernatant was appropriately diluted and filtered through a 0.22 μm aqueous filter membrane prior to HPLC injection and analysis. The HPLC system operated with a mobile phase of 5 mM H₂SO₄ solution at a flow rate of 0.6 mL/min, with a column temperature of 55 °C, a detector temperature of 35 °C, and an injection volume of 20 µL. The concentrations of intracellular NADH and NAD^+^ at different time points were measured using the Coenzyme I NAD(H) content assay kit (Beijing Solarbio Science & Technology Co., Ltd., BC0315) in accordance with the manufacturer’s instructions.

### Stoichiometric analysis and carbon balance

The formation of biomass, products, ATP, and NADH_2_ were stoicheiometrically described in Supplementary material. Briefly, the theoretical maximum and minimum NADH generation during co-metabolism were estimated by considering the minimum and maximum hydrogen production, respectively (Eqs. (1) and (2) ). The actual NADH consumption was calculated based on the experimentally quantified fermentation products, as shown in Eq. (3).

1 $$ \begin{aligned} {\text{Maximum NADH generation}} = & \\ & \left( {\left[ {{\text{Glycerol consumption}}} \right] - \left[ {{\mathrm{1}},{\mathrm{3}} - {\mathrm{PDO}}} \right]} \right) \times {\mathrm{3}} \\ & + \left[ {{\text{Glucose or Fructose consumption}}} \right] \times {\mathrm{4}} \\ \end{aligned} $$

2 $$ \begin{aligned} {\text{Minimum NADH generation}} = & \\ & \left( {\left[ {{\text{Glycerol consumption}}} \right] - \left[ {{\mathrm{1}},{\mathrm{3}} - {\mathrm{PDO}}} \right]} \right) \times {\mathrm{2}} \\ & + \left[ {{\text{Glucose or Fructose consumption}}} \right] \times {\mathrm{2}} \\ \end{aligned} $$

3 $$ \begin{aligned} {\text{NADH consumption}} = & \\ & \left[ {{\mathrm{1}},{\mathrm{3}} - {\mathrm{PDO}}} \right] + \left[ {{\text{Butyric acid}}} \right] \times {\mathrm{2}} \\ & + \left[ {{\text{Lactic acid}}} \right] \\ \end{aligned} $$

For carbon balance analysis, the linear equation between biomass (g/L) and OD_650_ value was shown as Eq. (4). Elemental composition of the biomass was represented by C_4_H_7_O_2_N, corresponding to a molecular weight of 101 g/mol. Carbon recovery was calculated based on experimentally detected metabolites and biomass, and expressed as the molar ratio of carbon recovery in products and biomass relative to consumed carbon during fermentation (Eq. (5)), where R_C_ denotes the carbon recovery.

4 $$\:\mathrm{B}\mathrm{i}\mathrm{o}\mathrm{m}\mathrm{a}\mathrm{s}\mathrm{s}=\frac{{\mathrm{O}\mathrm{D}}_{650}\times\:0.294}{101}$$

5 $${{\mathrm{R}}_{\mathrm{C}}}=\frac{{\left[ {1,3 - {\mathrm{PDO}}} \right]+\left[ {{\mathrm{Acetic~acid}}} \right]+\left[ {{\mathrm{Butyric~acid}}} \right]+\left[ {{\mathrm{Lactic~acid}}} \right]+\left[ {{\mathrm{Biomass}}} \right]}}{{\left[ {{\mathrm{Glycerol}}} \right]+\left[ {{\mathrm{Glucose~or~Fructose}}} \right]}}$$

### Statistical analysis

Statistical analysis was performed using Microsoft Excel software and the data obtained were expressed as mean ± standard deviation (SD). Assumptions of normality and homoscedasticity were tested using Shapiro-Wilk and Levene’s test, respectively. The Analysis of Variance (ANOVA) was used to determine the statistical significance of biological parameters in the experiments. Non-significant differences were indicated by ns and asterisks denoted statistically significant differences (* represents *P* < 0.05, ** *P* < 0.01, *** *P* < 0.001, and **** *P* < 0.001).

## Results

### Effects of sugar as co-substrate on glycerol metabolism

The shake-flask cultivations using sugar as co-substrate demonstrated that the ratio of glucose to glycerol significantly influenced the metabolic characteristics of the strain (Table S1). Moreover, the yield of 1,3-PDO exhibited a nonlinear response relationship with respect to the substrate ratio. To validate the scalability of shake flask results and investigate broader co-substrate adaptations, batch fermentations were performed in a 5.0 L bioreactor. The effects of four carbohydrates (glucose, xylose, fructose and L-arabinose) on glycerol metabolism were systematically investigated.

**Fig. 2:**
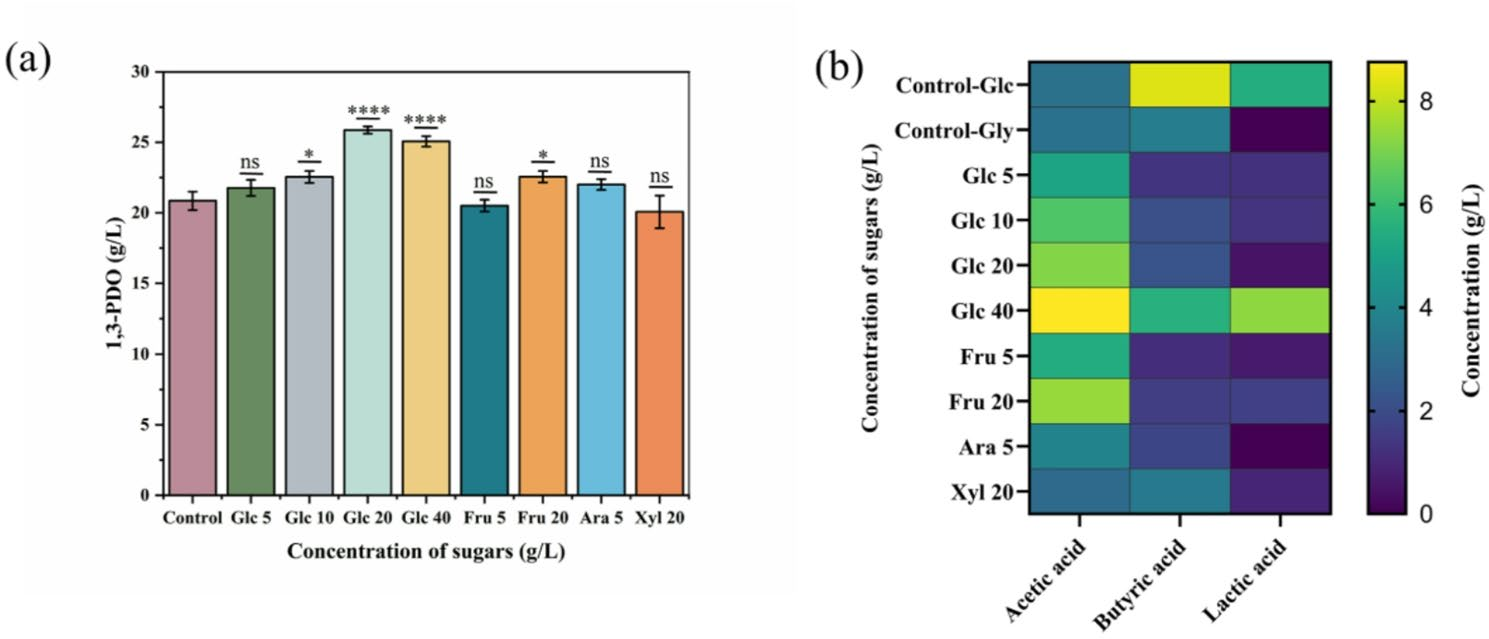
Product profiles in batch fermentations of glycerol and sugars. **a** Concentration of 1,3-PDO; **b** Concentration heatmap of acetic acid, butyric acid and lactic acid at different sugar concentrations. The number after abbreviated sugar represents its concentration (g/L)

Among the co-substrates tested, glucose and fructose enhanced glycerol metabolism, leading to increased 1,3-PDO production (Fig. 2a). The addition of glucose as a co-substrate had a significant effect on the metabolism of the *C. butyricum* DL07, especially when 20 g/L glucose was used as a co-substrate. Compared with glycerol as the sole carbon source, the concentration and yield of 1,3-PDO were observed to increase by 24.12% (25.88 g/L) and 24.24% (0.82 mol/mol), respectively (Fig. 2a; Table 3). Moreover, the highest yield (0.90 mol/mol) was achieved in a co-substrate fermentation at 40 g/L of glucose, at which a notable accumulation of by-products occurred, particularly acetic acid (8.76 g/L) and lactic acid (7.30 g/L) (Fig. 2b). Meanwhile, as shown in Table 3; Fig. 2, when glycerol was used as the sole carbon source at 40 g/L, it was completely consumed within 8 h, resulting in the production of 20.85 g/L 1,3-PDO, 3.23 g/L acetic acid, 3.67 g/L butyric acid, and no detectable lactic acid. When glucose was used as the sole carbon source at 40 g/L, 1,3-PDO was not observed and the metabolites were butyric acid, acetic acid, and lactic acid. And biomass (OD_650_) reached its maximum (15.11) in a short time (6 h). These results confirm that 1,3-PDO is produced by glycerol metabolism, and glucose has a significant effect on biomass and product distribution. Fructose as co-substrate also showed a synergistic effect with a 1,3-PDO yield from 0.66 to 0.81 mol/mol, but a reduction in productivity from 2.61 g/L/h to 1.88 g/L/h.

**Table 3:** The batch fermentation results of glycerol and different sugars

Fermentation time (h)	Co-substrate		Initial Glycerol (g/L)	Residual Sugar (g/L)	Residual Glycerol (g/L)	Biomass (max OD_650_)	Y_1,3−PDO_* (mol/mol gly)	Q_1,3−PDO_* (g/L/h)
	Sugar	Concentration (g/L)						
8	-	0	40	0	0	5.81 ± 0.26	0.66	2.61
6	Glc*	40	0		0	15.11 ± 0.15	0	0
12		5	40		0.64 ± 0.05	5.55 ± 0.42	0.75	1.81
10		10			1.55 ± 0.42	6.74 ± 0.38	0.81	2.26
10		20			0	12.16 ± 0.09	0.82	2.59
10		40			0	14.52 ± 0.18	0.90	2.51
12	Fru*	5			4.84 ± 0.03	5.15 ± 0.21	0.80	1.71
12		20			1.00 ± 0.26	7.46 ± 0.14	0.81	1.88
8	Ara*	5		4.91 ± 0.05	0	5.49 ± 0.13	0.66	2.75
12	Xyl*	20		20.01 ± 0.14	1.08 ± 0.08	3.88 ± 0.30	0.63	1.67

*Y_1,3−PDO_ represents the molar yield of 1,3-PDO to glycerol, Q_1,3−PDO_ represents the productivity of 1,3-PDO, Glc, Xyl, Fru and Ara are abbreviations of glucose, xylose, fructose, L-arabinose, respectively

In contrast, xylose and L-arabinose were not metabolized by *C. butyricum* DL07, and the concentration and yield of 1,3-PDO did not change significantly. However, it is interesting that 5 g/L L-arabinose resulted in the highest productivity (2.75 g/L/h), as well as a 20% increase in acetic acid concentration and a 50% decline in butyric acid concentration. These results exhibited that different sugars possess different influences on glycerol metabolism by *C. butyricum.*

### Genomic constraints on xylose and L-arabinose utilization

HPLC analysis showed that the residual concentrations of L-arabinose and xylose at the end of fermentation remained at 98.20 ± 1.00% and 100.05 ± 0.71% of their initial levels, respectively (Table 3). This observation stands in contrast to the complete consumption of glucose and fructose, indicating a potential defect in the pentose metabolic pathway of *C. butyricum* DL07. However, slight addition of L-arabinose resulted in an increase in acetic acid formation and a decline in butyric acid production. To investigate the genetic basis of this phenotype, the complete genome of *C. butyricum* DL07 (NCBI SRA Bank, SRR29209959) was compared with that of the classic strains, e.g. *Escherichia coli* MG1655 and *Bacillus subtilis* 168, which possess complete and functional xylose/L-arabinose metabolic modules. Additionally, *Clostridium diolis* DSM15410, a closely related strain reported to be capable of metabolizing xylose/L-arabinose and belonging to the same genus as *C. butyricum* DL07, was incorporated to account for phylogenetic relatedness.

**Fig. 3:**
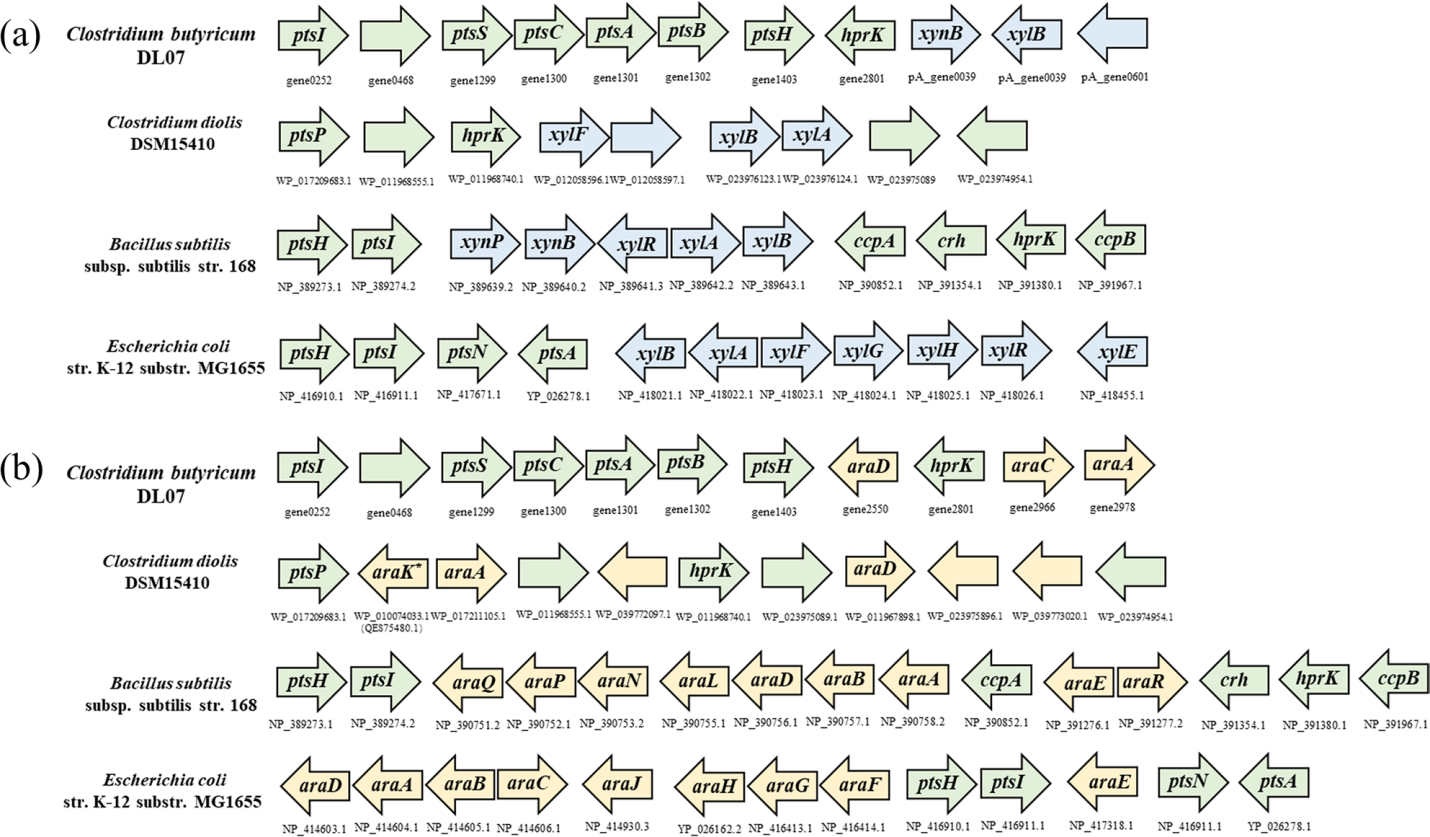
Summarization of chromosomal gene cluster for xylose **a** and L-arabinose **b** utilization in various strains

For xylose metabolism, comparative analysis demonstrated that the genome of *C. butyricum* DL07 specifically lacks key genes encoding catabolic enzymes and substrate transporters, such as *xylA*,* xylF*,* xylG*, and *xylH* (Fig. 3a) [37, 38]. The gene *xylA* encodes xylose isomerase (XI) that catalyzes the isomerization of D-xylose to D-xylulose as the rate-limiting step in prokaryotic xylose catabolism. XI is indispensable for initiating xylose assimilation via the PPP, as evidenced by complementary studies of xylose utilization restoration in XI deletion mutants [39]. The lack of subunits of a high-affinity xylose uptake system encoded by ATP-binding cassette (ABC) transporters (*xylF*, *xylG*, and *xylH*) genes also limits xylose utilization.

In the typical L-arabinose metabolic pathway, taking *E. coli* MG1655 and *B. subtilis* 168 as examples, the gene *araB* encodes L-ribulokinase, which is crucial for directing L-arabinose to the PPP. In contrast, certain *Clostridium* species may possess a nonorthologous replacement of AraB, termed AraK, which belongs to the FGGY sugar kinase family and performs the same catalytic function as AraB [40, 41]. However, neither the gene *araB* nor any gene encoding a protein similar to AraK is present in the genome of *C. butyricum* DL07. At the same time, the absence of *araF*, *araH*, and *araG*, which encode components of the ABC transporter, significantly impairs the uptake of L-arabinose (Fig. 3b). These genetic defects account for the inability of *C. butyricum* DL07 to metabolize L-arabinose.

It is noteworthy that despite the absence of the L-arabinose metabolic pathway, the genome of *C. butyricum* DL07 encodes multiple sugar-sensing and regulatory elements, such as *cre*, *rex*, and *ptsGHI*. Additionally, genes associated with stress response, redox regulation, and sporulation are retained, including *spo0A*, *sigH*, and *fnr*. This suggests that *C. butyricum* DL07 may reprogram its metabolic pathways in response to nutritional signals, and adapt to the presence of non-metabolizable sugars in the environment.

### Chronotropic transcriptional regulation and redox homeostasis

**Fig. 4:**
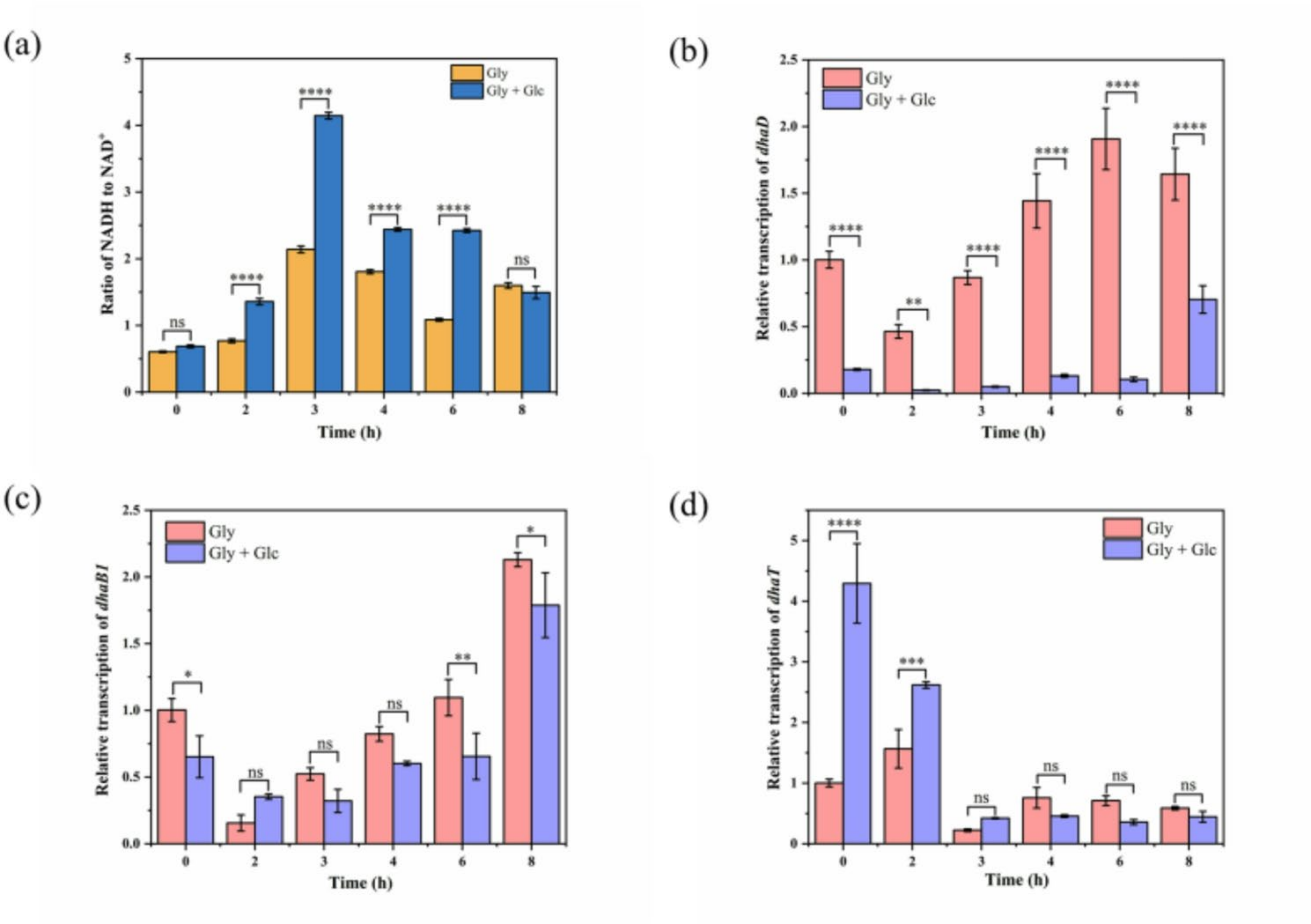
Effects of the glucose on transcriptional levels of key genes involved in glycerol metabolism and internal redox reducing equivalent during a batch fermentation. **a** Determination of intracellular NADH/NAD^+^ ratio. Relative transcriptional levels of **b ***dhaD*, **c ***dhaB1*, and **d** *dhaT* using real-time quantitative PCR

During assessing the effects of different combinations of carbon sources on the metabolic performance of the strain, it was found that NADH availability at different ratios was closely related to carbon flux distribution, affecting the ratio of fermentation products. During the co-metabolism of glucose/fructose and glycerol by *C. butyricum*, a stoichiometric representation of net reducing equivalents was applied to quantify NADH consumption. Accordingly, NADH demand was defined as 1 mol/mol 1,3-PDO, 2 mol/mol butyric acid, and 1 mol/mol lactic acid, respectively (Fig. 1). Based on the above, the NADH consumption was calculated from the actual batch fermentation products under anaerobic conditions (Table 4). By defining the theoretical maximum and minimum NADH generation through consideration of maximum and minimum hydrogen production, the NADH consumption was found to fall within this range, thereby supporting the rationality of NADH consumption in co-substrate metabolism. Consistently, based on simplified carbon balance calculation derived from experimentally measured product and biomass concentrations, the carbon recovery was consistently slightly above 100%, indicating that the overall carbon balance within the co-substrate metabolism is subject to reasonable constraints. As expected, the addition of glucose/fructose decreased the reliance on glycerol for NADH generation and reduced the production of butyric acid, a major NADH-consuming by-product, by 16.79–83.36%. Consequently, the concentration of the NADH-dependent product 1,3-PDO increased from 273.98 mM to 340.08 mM, and the carbon utilization efficiency from glycerol to 1,3-PDO increased from 65.55% to 82.60%. It is worth noting that the provision of NADH through glucose/fructose metabolism resulted in lactic acid formation, which could be maintained within a controllable range (4.77–81.04 mM) by adjusting the co-substrate ratio. Figure 4a shows the experimental verification results of the intracellular NADH/NAD^+^ ratio in actual batch fermentation between the control at initial glycerol concentration of 40 g/L and glucose co-substrate group at 20 g/L. As a result, the NADH/NAD^+^ ratio in the fermentation group using glucose as co-substrate was significantly higher than that in the single glycerol group by 1.35–2.23 folds, which is consistent with the effect of coupling of NADH-1,3-PDO synthesis as observed in *K. pneumoniae* [42].

**Table 4:** The NADH generation and consumption and carbon balance analysis using glucose or fructose as co-substrates in batch fermentations

Carbon source	Carbon consumed (mM)		Products (mM)				NADH consumption (mM)	NADH generation (mM)		NADH consumption rate (mmol/L/h)	Biomass (mM)	Molar yield of 1,3-PDO from Gly (%)	*R*_C_ (%)
	Gly	Sugar	1,3-PDO	Acetic acid	Butyric acid	Lactic acid		Min	Max				
Gly	417.95	0	273.98	53.83	75.70	0	425.38	287.34	431.01	53.17	16.91	65.55	100.59
Glc + Gly	379.66	24.41	286.07	84.76	14.98	13.65	329.69	235.99	378.40	27.47	16.16	75.35	102.86
	366.61	48.71	296.32	105.91	23.72	14.21	357.97	237.99	405.69	35.80	19.62	80.83	110.70
	411.71	105.23	340.08	119.07	24.97	4.77	394.79	353.72	635.80	39.48	35.40	82.60	101.42
	366.55	187.36	329.43	145.88	62.99	81.04	536.45	448.95	860.79	53.65	42.27	89.87	119.44
Fru + Gly	342.31	25.23	269.51	89.76	12.60	6.99	301.70	196.06	319.32	25.14	14.99	78.73	107.16
	366.84	96.53	296.45	123.73	17.93	18.87	351.19	333.85	597.30	29.27	21.72	80.81	103.31

**Fig. 5:**
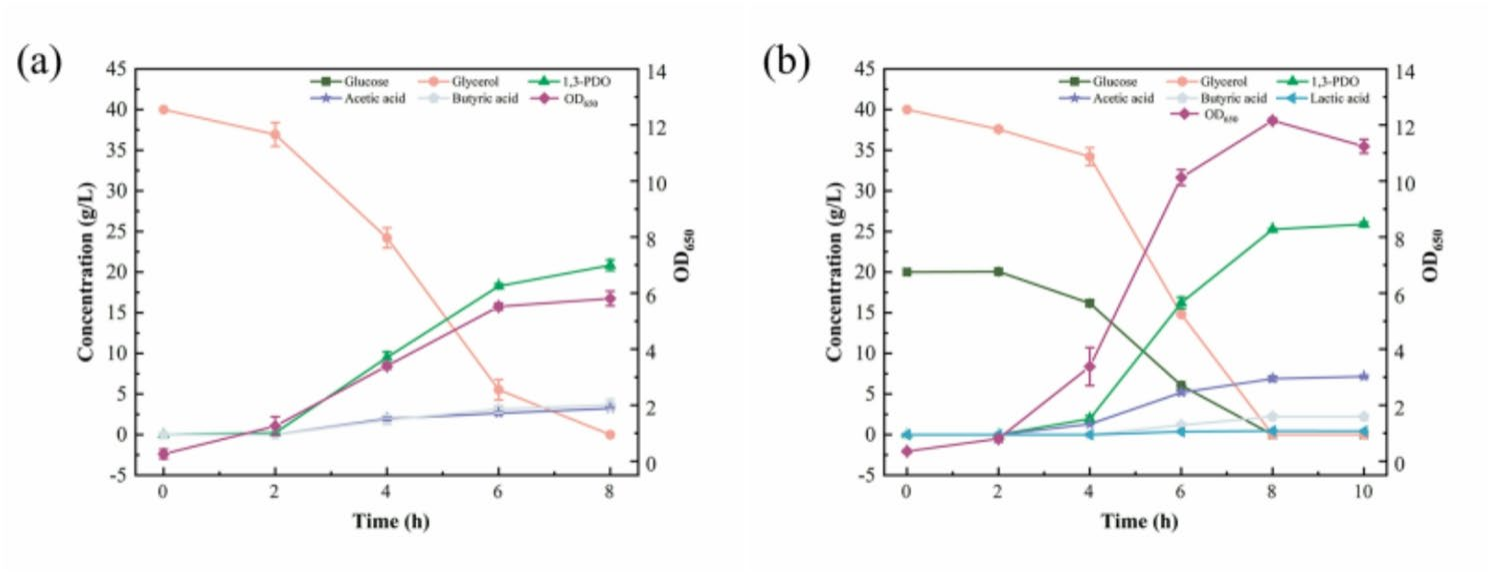
The batch fermentation results of **a** 40 g/L glycerol, and **b** 40 g/L glycerol and 20 g/L glucose

The balance between NADH and NAD^+^ actually reflected the metabolic flow direction towards NADH generation or consumption, thereby indicating alterations in metabolic pathways. The metabolic pathways are catalyzed by the corresponding enzymes, especially the related enzymes with 1,3-PDO production, i.e. glycerol dehydrogenase (GDH), 1,3-propanediol oxidoreductase (PDOR), and glycerol dehydratase (GDHt). The expression of enzyme depends on the mRNA level of its gene. The temporal transcriptional regulation of *dha* operon was investigated by real-time quantitative PCR analysis. When glucose was used as co-substrate, the mRNA level of *dhaD* (encoding GDH) showed significant suppression throughout fermentation, especially decreasing by more than 11.01 folds between 2 and 6 h (Fig. 4b). Correspondingly, the glycerol oxidation pathway was severely inhibited (Fig. 1). Interestingly, substantial biomass growth and accumulation of by-products were observed during this phase. The biomass (OD_650_) and acetic acid concentration in the glucose co-substrate group increased by 2.18 and 1.97 folds respectively. In contrast, the concentration of butyric acid decreased to 1.17 g/L, thereby reducing the consumption of reducing power and facilitating ATP production (Fig. 5). Therefore, it can be concluded that the addition of glucose significantly reduces the consumption of glycerol in the oxidation pathway, which is conducive to the production of 1,3-PDO. Furthermore, the expression of the two key enzymes involved in the glycerol reduction pathway for 1,3-PDO synthesis exhibited different regular changes. The *dhaB1* gene encoding GDHt was 59.78–83.98% of the control level at the beginning (0 h) and later (6–8 h) of fermentation, while *dhaT* (encoding PDOR) showed a significant 1.67–4.29 folds up-regulation in the early stage of fermentation (0–3 h) and then remained at a level comparable to the control (Fig. 4c, d). It should be emphasized that there is a significant time-lag between alterations in gene transcriptional levels and formation of products. Specifically, changes in mRNA levels of the glucose co-substrate group were observed during the early fermentation phase (0–3 h), whereas changes in substrates and products were detected during the 4–8 h period. These findings indicate that *C. butyricum* DL07 modulates its metabolic state by regulating the expression of the gene encoding the relevant enzyme, although this regulation appears to be indirect. This study provides the first evidence of temporal regulation of the *dha* operon in the metabolic synthesis of 1,3-PDO, establishing a framework for temporal optimization of co-substrate fermentation.

### Stoichiometric analysis of cometabolism

Stoichiometric equations were developed based on the metabolic pathway of glycerol in *C. butyricum* DL07 during fermentation using glucose as co-substrate in the supplementary material. These equations aim to clarify the theoretical constraints of carbon flux allocation and NADH regeneration. For simplicity, q is assumed here to be the molar ratio of biomass formed from glucose to total biomass, p is the molar ratio of pyruvic acid produced from glucose to total pyruvic acid, and α is the molar ratio of pyruvic acid catalyzed by pyruvate formate lyase (PFL) to total pyruvic acid, based on the previous work [34]. Similarly, β is the molar fraction of acetic acid in total acetyl-CoA metabolism, and then 1-β is the molar fraction of butyric acid. The yield of 1,3-PDO to glycerol (Y_PDO/Gly_) and the ratio of glucose to glycerol (R_Glc/Gly_) are expressed as follows.

6 $${{\mathrm{Y}}_{{\mathrm{PDO}}/{\mathrm{Gly}}}}=\frac{{{\mathrm{15(4-2p-\boldsymbol{\upalpha}-\boldsymbol{\upalpha}\boldsymbol{\upbeta}+2\boldsymbol{\upbeta})+2(3+\boldsymbol{\upbeta})(1-q)}}}}{{30\left( {1 - {\mathrm{p}}} \right)+4\left( {1 - {\mathrm{q}}} \right)\left( {3+{\mathrm{\boldsymbol{\upbeta}}}} \right)+15\left( {4 - 2{\mathrm{p}} - {\mathrm{\boldsymbol{\upalpha}}} - {\mathrm{\boldsymbol{\upalpha}\boldsymbol{\upbeta}}}+2{\mathrm{\boldsymbol{\upbeta}}}} \right)}}$$

7 $${{\mathrm{R}}_{{\mathrm{Glc}}/{\mathrm{Gly}}}}=\frac{{{\mathrm{15p+q(3+\boldsymbol{\upbeta})}}}}{{{\mathrm{30(1-p)+4(1-q)(3+\boldsymbol{\upbeta})+15(4-2p-\boldsymbol{\upalpha}-\boldsymbol{\upalpha}\boldsymbol{\upbeta}+2\boldsymbol{\upbeta})}}}}$$

Previous studies suggest that the ideal method to increase the yield of 1,3-PDO involves the exclusive production of acetic acid as a by-product [30]. This is indicated by Y_PDO/Gly_=0.72 mol/mol in the absence of glucose as co-substrate (*p* = 0, q = 0) and the absence of hydrogen and butyric acid formation (α = 0, β = 1).

Under the condition of α = 0, the yield of 1,3-PDO is described as the following equation with respect to R_Glc/Gly_ and β, according to Eqs. (6) and (7) by removing the parameters p and q:

8 $${{\mathrm{Y}}_{{\mathrm{PDO}}/{\mathrm{Gly}}}}=\frac{{{\mathrm{33+16\boldsymbol{\upbeta}+30(1+\boldsymbol{\upbeta})}}{{\mathrm{R}}_{{\mathrm{Glc/Gly}}}}}}{{{\mathrm{17(3+\boldsymbol{\upbeta})}}}}$$

Under the condition that the yield reaches a maximum at Y_PDO/Gly_=1 mol/mol and β = 1, the molar ratio of glucose to glycerol is R_Glc/Gly_=0.32 mol/mol.

**Fig. 6:**
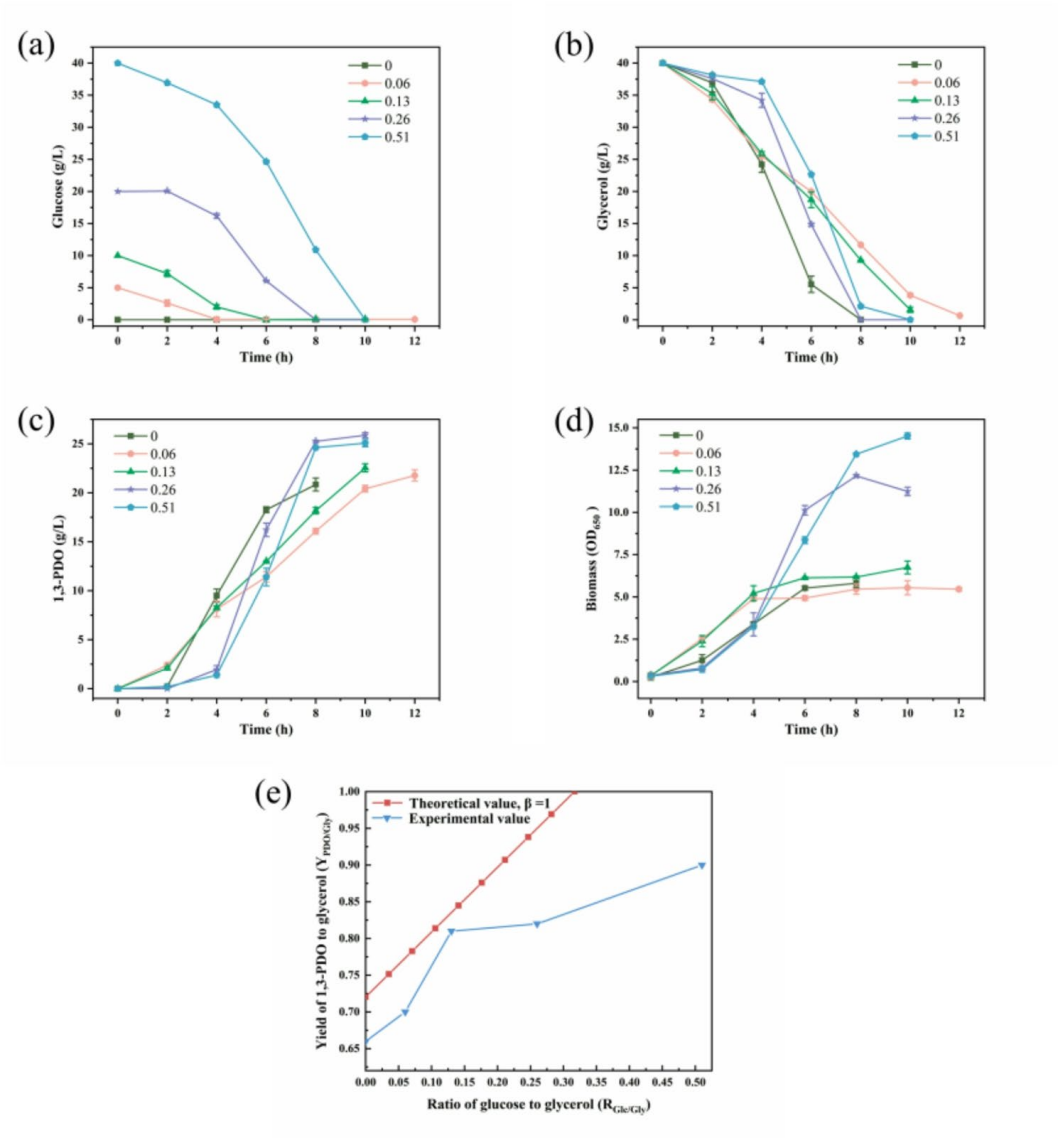
Batch fermentation curves at different ratios of glucose to glycerol. **a** Glucose, **b** Glycerol, **c** 1,3-PDO, **d** Biomass at R_Glc/Gly_=0, 0.06, 0.13, 0.26, 0.51 mol/mol. **e** Relationship between Y_PDO/Gly_ and R_Glc/Gly_

Under strict anaerobic conditions, batch fermentations were conducted by *C. butyricum* DL07 at initial R_Glc/Gly_ of 0, 0.06, 0.13, 0.26, and 0.51 mol/mol. As shown in Fig. 6a-b, glucose and glycerol were consumed simultaneously, but at different rates. Especially within 2–6 h, the presence of glucose reduced the glycerol consumption rate from 7.85 g/L/h to 3.58 g/L/h. Figure 6c and d respectively show that glucose supplementation promoted the accumulation of 1,3-PDO and biomass. However, compared with the control using glycerol as the sole carbon source, the fermentation time was also extended from 8 h to 12 h. Correspondingly, 1,3-PDO productivity declined from 2.61 g/L/h to 1.81 g/L/h in glucose-containing media.

In addition, compared with the theoretical yield, the experimental yield decreased under different ratios of glucose to glycerol (Fig. 6e). Firstly, as shown in Fig. 1, the enzymes lactate dehydrogenase (LDH), PFL, and pyruvate: ferredoxin oxidoreductase (PFOR) participate in pyruvate metabolism, leading to the generation of lactic acid, hydrogen, and acetyl-CoA. The generated acetyl-CoA is further converted into butyric acid, thereby reducing the availability of NADH for 1,3-PDO synthesis. Moreover, when glucose is supplied, the expression of GDH remains at a low level, which is insufficient to completely block the glycerol oxidation pathway (Fig. 4b), and part of the glycerol is also diverted toward biomass accumulation. Interestingly, when R_Glc/Gly_ is 0.13, Y_PDO/Gly_ approaches the theoretical yield most closely, although it does not achieve its maximum value. This phenomenon may be attributed to the relatively weak inhibitory effect of CCR resulting from the low concentration of glucose. Taken together, factors including carbon flux redistribution, energy demand, and environmental perturbations during actual fermentation processes collectively prevent the yield from reaching the theoretical maximum of 100%.

### Temporal feeding strategy based on metabolic analysis

**Fig. 7:**
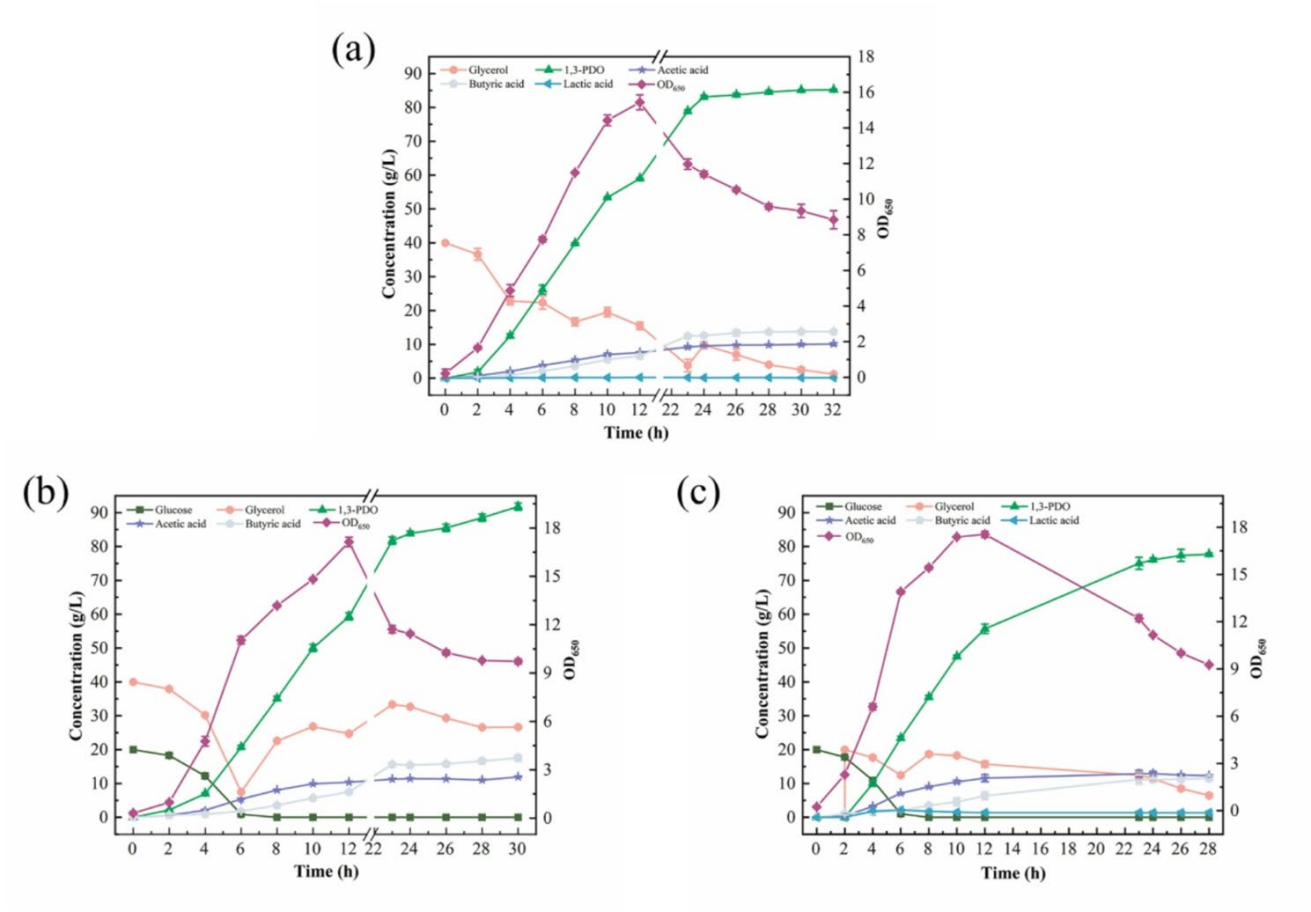
Fed-batch fermentation profiles using glucose as co-substrate. **a** Only glycerol as substrate; **b** 40 g glucose and glycerol added initially; **c** Feeding glycerol after 2 h when 40 g glucose added initially

Based on the key trade-off identified above between maximising 1,3-PDO yield and by-product control, the effectiveness of this approach was further validated by fed-batch fermentations with an initial fermentation volume of 2.0 L and optimized using temporal feeding strategies. As shown in Fig. 7a-b; Table 5, the initial substrate combination (40.0 g glucose + 80.0 g glycerol) significantly increased the 1,3-PDO yield to 0.76 mol/mol, as well as the 1,3-PDO concentration to 91.79 g/L. The by-products were dominated by butyric acid (17.54 g/L), without lactic acid detected, indicating that the carbon flux was preferentially directed towards NADH-dependent 1,3-PDO synthesis. This result directly demonstrates the effectiveness of stoichiometric analysis in guiding co-substrate fermentation design.

**Table 5:** Comparison among the fed-batch fermentations using different feeding strategies

Glucose (g)	Time (h)	1,3-PDO (g/L)	Acetic acid (g/L)	Butyric acid (g/L)	Lactic acid (g/L)	Y_1,3−PDO_ (mol/mol gly)	Y_1,3−PDO_ (g/g gly + glc)	Q_1,3−PDO_ (g/L/h)
0	32	85.20 ± 0.25	10.11 ± 0.39	13.80 ± 0.19	0.17 ± 0.03	0.63	0.52	2.66
40.0^a^	30	91.79 ± 1.09	11.94 ± 0.11	17.54 ± 0.95	0	0.76	0.57	3.06
40.0^b^	28	77.70 ± 0.37	12.31 ± 0.27	11.51 ± 0.71	1.39 ± 0.45	0.72	0.53	2.78
35.8^c^	26	83.50 ± 1.09	9.34 ± 0.11	14.80 ± 0.64	2.45 ± 0.02	0.73	0.55	3.21
39.0^d^	26	84.15 ± 0.12	9.97 ± 0.02	14.28 ± 1.09	3.59 ± 0.07	0.74	0.55	3.24
83.0^c^	24	71.88 ± 2.18	10.68 ± 0.44	13.74 ± 0.92	6.04 ± 0.60	0.74	0.49	3.00
89.2^d^	24	68.38 ± 2.45	11.28 ± 0.38	12.35 ± 0.70	5.74 ± 0.06	0.86	0.54	2.85
83.9^e^	23	69.12 ± 0.84	11.89 ± 0.36	11.05 ± 1.38	8.07 ± 0.06	0.74	0.49	3.01

Types of glucose and glycerol addition: ^a^Adding glucose and glycerol at the initial stage of fermentation

^b^Adding glucose at the initial stage of fermentation and feeding glycerol after 2 h fermentation

^c^Adding glycerol at the initial stage of fermentation and feeding glucose at a constant rate in 6 h during initial fermentation

^d^Adding glycerol at the initial stage of fermentation and feeding glucose at a constant rate in 10 h during initial fermentation

^e^Adding glycerol and glucose (40 g) at the initial stage of fermentation and feeding glucose (43.9 g) during fermentation

Actually, theoretical analyses did not account for the effects of time-dependent metabolic regulation. Fed-batch fermentations demonstrated that the feeding time of glucose is critical for NADH supply and carbon flow allocation. As shown in Fig. 7c, supplying only 40.0 g glucose at the beginning of fermentation could shorten the lag period, but the delayed feeding of glycerol after 2 h would lead to the inhibition of gene expression in glycerol metabolism, and decreased the yield of 1,3-PDO from 0.76 to 0.72 mol/mol. This phenomenon highlighted the need for synchronized supply of substrate and co-substrate, and indicated the necessity of integrating theoretical analysis with temporal metabolic regulation to enhance prediction accuracy.

Further investigation of various feeding strategies uncovered the dual role of NADH supply in metabolic flux control. As shown in Table 5, fermentation performance exhibited marked dependence on glucose feeding strategy, resulting in distinct metabolic results among feeding strategies that use the same glucose mass (40/80 g) but employ different supplementation protocols. On the other hand, it was found that high levels of glucose (> 80 g), while enhancing the supply of reducing power, induced acetic acid accumulation and decreased 1,3-PDO concentration, which are consistent with the reports about metabolic inhibitory effect [43, 44]. This was also accompanied by increased lactic acid accumulation, which further enhanced the metabolic inhibitory effect. Notably, the yield reached 0.86 mol/mol after 10 h of 89.2 g glucose supplementation. To our knowledge, this was the highest 1,3-PDO yield ever achieved in a fed-batch fermentation of glycerol by a natural strain using glucose as co-substrate. In addition, the highest productivity of the fed-batch fermentation was 3.24 g/L/h under the condition of feeding 39.0 g glucose over 10 h. Therefore, the existence of a threshold effect of the glycerol/glucose ratio on NADH supply must be circumvented to avoid by-product formation and enhance the target yield.

### Engineering significance

Co-substrate engineering regulates metabolic flux allocation by introducing auxiliary carbon sources, which becomes a beneficial means to optimize the efficiency of reducing power supply and carbon flux. According to previous reports, 1,3-PDO produced through co-substrate metabolic engineering has been used in the fermentation of various strains (Table 6). Unfortunately, previous studies have generally been constrained by the low concentration or yield of 1,3-PDO. In contrast, this study addresses this limitation by achieving high yield of 1,3-PDO at elevated concentrations. Given the substantial price difference between glycerol ($500–700/t) and glucose ($200–300/t), co-fermentation of glucose and glycerol significantly reduces the total cost of raw material for 1,3-PDO. For instance, the results of the present study demonstrate that a molar ratio of glucose to glycerol being 0.16 increases the yield from 0.63 to 0.86 mol/mol, which results in reduction of the raw material cost by 13–20%. Therefore, it can be concluded that co-substrate metabolism engineering in *C. butyricum* DL07 offers a promising strategy with enhanced industrial potential for efficient 1,3-PDO production.

**Table 6:** Comparison of 1,3-PDO production by different natural microorganisms using sugar as co-substrate

Microorganism	Co-substrate	Fermentation mode	C_1,3−PDO_ (g/L)	Y_1,3−PDO_ (mol/mol gly)	Q_1,3−PDO_ (g/L/h)	Reference
*C. butyricum* L4	Glc	Flask	-	0.88	1.32	[5]
*C. butyricum* DSM 5431	Glc	Flask	15.22	0.92–0.93	-	[45]
*K. Pneumoniae* ME-303	Xyl	Flask	10.35	0.42	0.36	[32]
	Mannose	Flask	9.79	0.39	0.34	
	Hemicellulosic hydrolysates	Fed-batch	71.58	0.65	1.93	
*C. diolis* DSM 15,410	Glc	Batch	14.70	0.86	1.13	[41]
	Corn stover hydrolysate	Batch	14.58	0.85	-	
		Fed-batch	43.5	0.82	1.4	
*C. butyricum* E5	Glc	Batch	6.36	0.89	-	[46]
*C. Beijerinckii* CICC 22,954	Ara	Batch	21.6	0.87	0.45	[47]
	Glc	Batch	23.3	0.94	0.49	
*C. butyricum* DL07	Fru	Batch	22.56	0.81	1.88	This study
	Glc	Batch	25.07	0.90	2.51	This study
	Glc	Fed-batch	91.79	0.76	3.06	This study

## Discussion

Co-substrate fermentation has been shown to be one of the effective ways to increase the concentration and yield of target products. In this study, the batch fermentations using sugar as co-substrate including glucose, fructose, xylose and L-arabinose were carried out to evaluate the role of different sugars in increasing the yield of 1,3-PDO. Glucose was proved to be the optimal co-substrate, where a significant increase in metabolic flux towards 1,3-PDO synthesis was observed, and glycerol was completely consumed. This was due to the redistribution of carbon metabolic fluxes under high glycolytic activity.

The addition of glucose was effective in alleviating the redox imbalance during glycerol metabolism, although the increase in 1,3-PDO yield was at the expense of by-product production, such as acetic acid and lactic acid. It is worth noting that the formation of lactic acid represents an adaptive mechanism by which *C. butyricum* DL07 maintains intracellular NADH/NAD^+^ balance. While glucose metabolism relieves the NADH limitation imposed on the reductive branch of glycerol metabolism, an excessive glycolytic flux may result in surplus NADH, which is partially reoxidized through the lactate synthesis pathway to restore redox equilibrium. Remarkably, solvent products (such as butanol and ethanol) were not detected in the glucose/glycerol co-substrate fermentation system using *C. butyricum* DL07. This metabolic phenotype minimized carbon flux diversion and, critically, simplified downstream product isolation and purification.

In contrast, the co-substrate performance of fructose was lower than glucose, potentially due to transport efficiency limitations in the fructose phosphotransferase system (PTS). In addition, xylose and L-arabinose were not utilized by *C. butyricum* DL07, but did affect fermentation. This phenomenon suggests that non-metabolizable carbon sources may indirectly modulate metabolic networks through bypass regulations [48]. L-Arabinose could potentially induce a metabolic stress response via CCR, prompting *C. butyricum* DL07 to enhance glycerol catabolism for competitive carbon source utilization [49, 50]. This metabolic shift upregulates key enzyme activities, including GDHt and PDOR, thereby increasing carbon flux toward 1,3-PDO production. The concomitant metabolic reorganization maintains redox homeostasis, with the strain compensating through NADH-independent acetic acid synthesis to preserve cofactor balance.

Genomic information revealed that *C. butyricum* DL07 lacks genes encoding xylose isomerase and L-ribulokinase or enzymes performing the same function, which is a major reason for its inability to metabolise xylose and L-arabinose. The absence of specific transporters could also limit intracellular xylose and L-arabinose accumulation, failing to meet the threshold required for metabolic flux. In conclusion, the xylose and L-arabinose pathways are ‘metabolically redundant’ owing to the absence of substrate selection pressure in the external and genetic environment. Accumulated mutations in these non-essential genes were fixed by the lack of purifying selection, resulting in gene loss [51].

An in-depth investigation of the metabolic mechanism of co-substrate fermentation revealed that glucose metabolism influences the direction of pyruvate node metabolism by modulating the NADH/NAD^+^ balance (Fig. 1), which is manifested by an increase in the NADH/NAD^+^ ratio to promote glycerol reductive metabolism for the synthesis of 1,3-PDO. Meanwhile, cometabolism showed stage-specific regulation of key genes in glycerol metabolism, which was closely related to the prioritization of carbon source metabolism. Fructose-1,6-bisphosphate (FBP) is typically produced during glucose metabolism, which activates HPr kinase/phosphatase (HPrK/P). The activated HPrK/P subsequently forms a complex with catabolite control protein A (CcpA), which binds to catabolite-responsive element (*cre*) sites in target genes, resulting in transcriptional repression. In *C. butyricum* DL07, the promoter regions of *dhaD* and *dhaB1* were predicted to harbor weakly conserved *cre* sites, suggesting a potential for low-affinity CcpA binding and regulation, which is stage-specific or condition-dependent. Moreover, variations in *cre* sequences may influence the binding affinity of CcpA and thus alter the sensitivity of target genes to CCR. Hence, *dhaD*, *dhaB1*, and *dhaT* exhibit different patterns of transcriptional patterns in the presence of glucose. This study reveals the temporal regulation of the *dha* operon in *C. butyricum* DL07, establishing a framework for optimization of co-substrate fermentation.

Stoichiometric analysis of glycerol and glucose cometabolism in *C. butyricum*, based on energy, reducing equivalents, and biomass balance, suggests that the fermentation were carried out at a co-substrate ratio of 0.32 mol glucose/mol glycerol, and glycerol would be completely converted to 1,3-PDO. Meanwhile, the fed-batch fermentations guided by stoichiometric analysis revealed the importance of the feeding strategy (time and mode in Table 5), which served as a benchmark for subsequent feeding optimization. Optimization of the glucose/glycerol molar ratio balanced the regeneration of NADH from glucose catabolism with the efficiency of glycerol conversion, thereby alleviating the metabolic burden of the oxidative pathway (the NAD⁺-dependent GDH). As a result, maintaining a low molar ratio of glucose to glycerol (0.1–0.2) is optimal for facilitating the synthesis of 1,3-PDO. In contrast, a high molar ratio of glucose to glycerol may intensify the CCR effect on 1,3-PDO production and lead to the accumulation of by-products. This mechanism provides sufficient NADH for 1,3-PDO synthesis while maintaining cell growth. For example, the addition of glucose significantly increased the 1,3-PDO yield of *C. butyricum* DSM 5431 from 0.57 mol/mol to 0.92–0.93 mol/mol, representing a 60% increase [45]. This metabolic flexibility highlights the critical role of co-substrate engineering in overcoming the reductive power limitation present in the reduction pathway for 1,3-PDO formation in sole glycerol fermentation systems. Meanwhile, the material cost analysis demonstrated that the co-substrate strategy could effectively improve the economics compared to the sole glycerol system. Additionally, previous studies on co-substrate engineering for 1,3-PDO production have reported that lignocellulosic hydrolysates, when employed as co-substrates, markedly enhance product synthesis (Table 6). Given the low cost, accessibility, and renewability of lignocellulosic resources, together with the high conversion efficiency of *C. butyricum* DL07 toward crude glycerol, the use of thes as co-substrate for fermentation presents a promising future perspective.

*C. butyricum* DL07 among the natural strains producing 1,3-PDO has exihibited a promising robustness for industrial applications. In order to reduce cost for 1,3-PDO production, the rational feeding strategies, high target product concentration, yield and productivity are highly desirable [52]. The synergistic utilization of sugar and glycerol significantly enhanced the efficiency of 1,3-PDO biosynthesis by redirecting metabolic fluxes towards the reductive pathway [53]. However, scale-up challenges such as maintaining the stability of the strain culture and optimizing gas exchange in anaerobic fermentation still need to be thoroughly investigated to ensure the economic viability of industrial conversion. While the actual engineering results may not meet the expectations of this study, the time series-based co-substrate feeding strategy remains promising for fermentation engineering. In the future, incorporating dynamic feeding rates into metabolic flux models could further reveal the dynamic NADH supply-demand balance, enhance the accuracy of stoichiometric analysis models, and unlock the potential of sustainable biomanufacturing.

## Conclusion

In this study, the metabolic regulation of the glucose-glycerol cometabolism to produce 1,3-PDO by *C. butyricum* DL07 and its industrial application potential were systematically elucidated. Firstly, genomic analysis revealed that *C. butyricum* DL07 is unable to metabolize two pentoses, i.e. xylose and L-arabinose, due to the absence of key metabolic enzyme genes (*xylA*,* xylFGH*,* araB/K*, and *araFGH*). Through screening and optimization, the yield of 1,3-PDO was increased to 0.90 mol/mol at a glucose/glycerol molar ratio of 0.51, representing a 36.36% increase compared to the glycerol-only system. Analysis of the metabolic mechanism revealed that low glucose concentration supplemented NADH via glycolysis and alleviated the redox imbalance, whereas excess glucose inhibited glycerol metabolism and induced the accumulation of by-products via the CCR effect. Concurrently, the addition of glucose can effectively regulate *dha* operon expression, inhibiting GDH (*dhaD*) and phasically activating PDOR (*dhaT*). A temporal feeding strategy based on stoichiometric analysis increased the concentration of 1,3-PDO up to 91.79 g/L. Alternatively, the yield could be increased to 0.86 mol/mol, which represents the highest level of fed-batch fermentation reported to date for natural strains, with a concomitant 13–20% reduction in raw material cost. This study provides theoretical support and a scalable process scheme for the efficient biomanufacturing of 1,3-PDO, and lays the foundation for the application of temporal metabolic regulation strategies in industrial biotechnology.

## Supplementary Information

Below is the link to the electronic supplementary material.

Supplementary Material 1.

## Data Availability

The datasets generated and analyzed during the current study are available from the corresponding author upon reasonable request.

## Abbreviations

1,3-PDO: 1,3-Propanediol
Gly: Glycerol
Glc: Glucose
Fru: Fructose
Xyl: Xylose
Ara: L-Arabinose
PPP: Pentose phosphate pathway
PTT: Polytrimethylene terephthalate
CAGR: Compound annual growth rate
CCR: Carbon catabolite repression
OD: Optical density
HPLC: High-performance liquid chromatography
SD: Standard deviation
ANOVA: Analysis of Variance
XI: Xylose isomerase
ABC transporter: ATP-binding cassette transporter
GDH: Glycerol dehydrogenase
GDHt: Glycerol dehydratase
PDOR: 1,3-propanediol oxidoreductase
PFL: Pyruvate formate lyase
LDH: Lactate dehydrogenase
PFOR: Pyruvate:ferredoxin oxidoreductase
Y_PDO/Gly_: Yield of 1,3-PDO to glycerol
R_Glc/Gly_: Ratio of glucose to glycerol
PTS: Phosphotransferase system
FBP: Fructose-1,6-bisphosphate
HPrK/P: HPr Kinase/Phosphorylase
HPr: Histidine phosphocarrier protein
CcpA: Catabolite control protein A
Cre: Catabolite-responsive element
