# Supplementary material for: Cometabolic regulation of glucose and glycerol to enhance 1,3-propanediol yield by Clostridium butyricum
Source: Microb Cell Fact. 2026 Mar 8;25:97. doi: 10.1186/s12934-026-02971-6 (PMC13081559; doi:10.1186/s12934-026-02971-6)
Supplement: Supplementary file 1 — Supplementary Material 1. [file 12934_2026_2971_MOESM1_ESM.docx]

# **Supplementary Information**

# **Cometabolic regulation of glucose and glycerol to enhance 1,3-propanediol yield by *Clostridium butyricum***

Li Wang^1^, Yaqin Sun^1^ & Zhilong Xiu^1*^

^1^ MOE Key Laboratory of Intelligent Bio-Manufacturing, School of Bioengineering, Dalian University of Technology, Dalian 116024, China; [wl17854115755@163.com](mailto:wl17854115755@163.com) (L.W.); [sunyaqin@dlut.edu.cn](mailto:sunyaqin@dlut.edu.cn) (Y.S.)

^*^ Correspondence: [zhlxiu@dlut.edu.cn](mailto:zhlxiu@dlut.edu.cn)

Table S1 The shake-flask fermentation results of 40 g/L glycerol and glucose as co-substrates

| Glucose (g/L) | Residual glycerol  (g/L) | Product (g/L) | | | | Y_1,3-PDO_  (mol/mol gly) |
| --- | --- | --- | --- | --- | --- | --- |
|  |  | 1,3-PDO | Acetic acid | Butyric acid | Lactic acid |  |
| 0 | 13.87 | 14.01 | 1.47 | 1.56 | 0.66 | 0.56 |
| 10 | 30.56 | 7.11 | 1.75 | 1.88 | 3.01 | 0.62 |
| 5 | 28.80 | 7.90 | 1.77 | 1.82 | 1.44 | 0.61 |
| 3.33 | 26.50 | 9.34 | 1.68 | 1.38 | 1.62 | 0.63 |
| 2.5 | 26.59 | 9.20 | 1.60 | 1.62 | 0.53 | 0.63 |
| 2 | 26.75 | 9.67 | 1.54 | 1.47 | 0.97 | 0.67 |

# **Stoichiometric analysis**

The nomenclature used in the study is defined as follows：

Table S2 Nomenclature and definitions of symbols

| Symbol | Description |
| --- | --- |
| Y_ATP_ | the energetic yield of the biomass was taken as 10.1 g/mol |
| q | molar ratio of biomass formed from glucose to total biomass |
| 1−q | molar ratio of biomass formed from glycerol to total biomass |
| α | molar ratio of pyruvic acid catalyzed by PFL to total pyruvic acid |
| 1−α | molar ratio of pyruvic acid catalyzed by pyruvate:ferredoxin oxidoreductase (PFOR) to total pyruvic acid |
| β | molar fraction of acetic acid in total acetyl-CoA metabolism |
| 1-β | molar fraction of butyric acid in total acetyl-CoA metabolism |
| p | molar ratio of pyruvic acid produced from glucose to total pyruvic acid |
| 1-p | molar ratio of pyruvic acid produced from glycerol to total pyruvic acid |

C_4_H_7_O_2_N was used to denote the elemental composition of the biomass, corresponding to a molecular weight of 101 g/mol. The formation of biomass from glycerol and glucose can be written as follows:

4C_3_H_8_O_3_+3NH_3_+3$\frac{\mathrm{MW}}{Y_{\mathrm{ATP}}}$ATP→3C_4_H_7_O_2_N+4NADH_2_+6H_2_O (1a)

2C_6_H_12_O_6_+3NH_3_+3$\frac{\mathrm{MW}}{Y_{\mathrm{ATP}}}$ATP→3C_4_H_7_O_2_N+6H_2_O (1b)

The above equations were simplified as follows, respectively.

4C_3_H_8_O_3_+3NH_3_+30ATP→3C_4_H_7_O_2_N+4NADH_2_+6H_2_O (2a)

2C_6_H_12_O_6_+3NH_3_+30ATP→3C_4_H_7_O_2_N+6H_2_O (2b)

The total equation of biomass formation can be expressed as follows:

4(1-q)C_3_H_8_O_3_+2qC_6_H_12_O_6_+3NH_3_+30ATP→3C_4_H_7_O_2_N+4(1-q)NADH_2_+6H_2_O (3)

The metabolism of glucose or glycerol to pyruvic acid (C_3_H_4_O_3_) can be described by the following stoichiometric equations (4a) and (4b), respectively.

C_6_H_12_O_6_→2ATP+2NADH_2_+2C_3_H_4_O_3_ (4a)

C_3_H_8_O_3_→ATP+2NADH_2_+C_3_H_4_O_3_ (4b)

From Fig. 1, the cleavage of pyruvic acid to acetyl-CoA is catalyzed by two enzymes, PFL and PDOR. The equations for the formation of acetic acid and butyric acid from pyruvic acid, without considering the formation of lactic acid, are as follows:

C_3_H_4_O_3_+H_2_O→C_2_H_4_O_2_+ATP+(1-α)NADH_2_+CO_2_+αH_2_ (5)

C_3_H_4_O_3_+$\frac{\alpha}{2}$NADH_2_→$\frac{1}{2}$C_4_H_8_O_2_+$\frac{1}{2}$ATP+CO_2_+$\frac{\alpha}{2}$H_2_ (6)

Equations (7)-(9) can be obtained by definition.

α=$\frac{\left[ \mathrm{Formic} \mathrm{acid} \right]+\left[ H_{2} \right]}{\left[ \mathrm{Butyric} \mathrm{acid} \right]+\left[ \mathrm{Acetic} \mathrm{acid} \right]}$ (7)

β=$\frac{\left[ \mathrm{Acetic} \mathrm{acid} \right]}{\left[ \mathrm{Butyric} \mathrm{acid} \right]+\left[ \mathrm{Acetic} \mathrm{acid} \right]}$ (8)

1-β=$\frac{\left[ \mathrm{Butyric} \mathrm{acid} \right]}{\left[ \mathrm{Butyric} \mathrm{acid} \right]+\left[ \mathrm{Acetic} \mathrm{acid} \right]}$ (9)

The total pyruvic acid reaction catalyzed by PFOR is composed of two pathways: acetic acid and butyric acid. It can be summarized by the following computation: β×(5)+(1-β)×(6):

C_3_H_4_O_3_→βC_2_H_4_O_2_+$\frac{(1-\beta)}{2}$C_4_H_8_O_2_+CO_2_+$\frac{(1+\beta)}{2}$ATP+$\frac{(2\beta-\alpha\beta-\alpha)}{2}$NADH_2_

+$\frac{(\alpha\beta+\alpha)}{2}$H_2_-βH_2_O (10)

Then the total stoichiometric equation of glucose and glycerol oxidation can be described by [(4a)×p+(4b)×2(1−p)+(10)×2]×3:

3pC_6_H_12_O_6_+6(1-p)C_3_H_8_O_3_→6βC_2_H_4_O_2_+3(1-β)C_4_H_8_O_2_+6CO_2_+3(3+β)ATP

+3(4-2p-α-αβ+2β)NADH_2_+3(αβ+α)H_2_-6βH_2_O (11)

The production of 1,3-PDO can be described by equation (12).

C_3_H_8_O_3_+NADH_2_→C_3_H_8_O_2_+H_2_O (12)

Ideally, the energy produced by the oxidative pathway is consumed for the formation of biomass and reducing equivalents in the reducing pathway of glycerol metabolism to produce 1,3-PDO. Therefore, a balance equation of ATP equation can be described by (3+β)×(3)+10×(11):

[30p+2q(3+β)]C_6_H_12_O_6_+[60(1-p)+4(1-q)(3+β)]C_3_H_8_O_3_+3(3+β)NH_3_→

3(3+β)C_4_H_7_O_2_N+60βC_2_H_4_O_2_+30(1-β)C_4_H_8_O_2_+60CO_2_+30(αβ+α)H_2_+(18-54β)H_2_O

+[30(4-2p-α-αβ+2β)+4(3+β)(1-q)]NADH_2_ (13)

Based on the NADH_2_ balance, the total stoichiometric equation consisting of oxidative, reductive, and biosynthesis pathways can be obtained as (13)+[30(4-2p-α-αβ+2β)+4(3+β)(1-q)]×(12):

[30p+2q(3+β)]C_6_H_12_O_6_+[60(1-p)+8(1-q)(3+β)+30(4-2p-α-αβ+2β)]C_3_H_8_O_3_

+3(3+β)NH_3_→3(3+β)C_4_H_7_O_2_N+60βC_2_H_4_O_2_+30(1-β)C_4_H_8_O_2_+60CO_2_+30(αβ+α)H_2_

+[30(4-2p-α-αβ+2β)+4(3+β)(1-q)+18-54β]H_2_O+[30(4-2p-α-αβ+2β)

+4(3+β)(1-q)]C_3_H_8_O_2_ (14)
